# Exploring Mechanisms of Action: Using a Testing Typology to Understand Intervention Performance in an HIV Self-Testing RCT in England and Wales

**DOI:** 10.3390/ijerph17020466

**Published:** 2020-01-10

**Authors:** T. Charles Witzel, Peter Weatherburn, Adam Bourne, Alison J. Rodger, Chris Bonell, Mitzy Gafos, Roy Trevelion, Andrew Speakman, Fiona Lampe, Denise Ward, David T. Dunn, Michelle M. Gabriel, Leanne McCabe, Justin Harbottle, Yolanda Collaco Moraes, Susan Michie, Andrew N. Phillips, Sheena McCormack, Fiona M. Burns

**Affiliations:** 1 Department of Public Health, Environments and Society, London School of Hygiene and Tropical Medicine, London WC1H 9SH, UK; peter.weatherburn@lshtm.ac.uk (P.W.); chris.bonell@lshtm.ac.uk (C.B.); 2 Australian Research Centre in Sex, Health and Society, La Trobe University, Melbourne 3086, Australia; a.bourne@latrobe.edu.au; 3 Institute for Global Health, University College London, London NW3 2PF, UK; Alison.rodger@ucl.ac.uk (A.J.R.); a.speakman@ucl.ac.uk (A.S.); f.lampe@ucl.ac.uk (F.L.); andrew.phillips@ucl.ac.uk (A.N.P.); f.burns@ucl.ac.uk (F.M.B.); 4 Department of Global Health and Development, Faculty of Public Health and Policy, London School of Hygiene and Tropical Medicine, London WC1h 9SH, UK; mitzy.gafos@lshtm.ac.uk; 5 HIV i-Base, London SE1 3LJ, UK; roy.trevelion@i-base.org.uk; 6 Medical Research Council Clinical Trials Unit, University College London, London, WC1V 6LJ, UK; denise.ward@ucl.ac.uk (D.W.); d.dunn@ucl.ac.uk (D.T.D.); m.gabriel@ucl.ac.uk (M.M.G.); leanne.mccabe@ucl.ac.uk (L.M.); y.collaco-moraes@ucl.ac.uk (Y.C.M.); s.mccormack@ucl.ac.uk (S.M.); 7 SH:24, London SE1 7JB, UK; Justin@SH24.org.uk; 8 Centre for Behaviour Change, University College London, London WC1N 3AZ, UK; s.michie@ucl.ac.uk

**Keywords:** HIV testing, self-testing, men who have sex with men, COM-B, evaluation

## Abstract

SELPHI involves two interventions: A provides one HIV self-testing (HIVST) kit; B offers 3-monthly repeat HIVST kits if participants report ongoing risk. A logic model underpinned by the Behaviour Change Wheel informed the design of the intervention. SELPHI recruited 10,135 cis-men and trans people in England and Wales, all reporting anal sex with a man. This paper explores how the interventions were experienced and the mechanisms of action leading to impact for different groups of trial participants. In-depth interviews with 37 cis-men who have sex with men (MSM) were used to inductively categorise participants based on sexual and HIV testing histories. Themes relating to intervention experiences and impacts were mapped onto SELPHI-hypothesised intermediate outcomes to consider intervention impacts. Three groups were identified: ‘inexperienced testers’ engaged with SELPHI to overcome motivational and social and physical opportunity testing barriers. For ‘pro self-testers’, testing frequency was constrained by psychological and social barriers and lack of opportunity. ‘Opportunistic adopters’ engaged in HIVST for novelty and convenience. Perceived impacts for inexperienced testers were most closely aligned with the logic model, but for opportunistic adopters there was little evidence of impact. Distinctive groups were discernible with divergent intervention experiences. Using COM-B as a model for understanding behaviour change in relation to HIVST, our results indicate how HIVST interventions could be adapted to respond to different needs based on the target population’s demographic and behavioural features.

## 1. Introduction

### 1.1. HIV Self-Testing in the UK

Early HIV diagnosis ensures improved treatment outcomes and a definitive body of literature demonstrates the impossibility of sexual transmission of HIV once an undetectable viral load has been achieved [1,2,3,4,5]. In response to this, HIV testing has undergone a rapid evolution over the last two decades, with increases in the variety of testing options offered in many settings [6,7]. Conceptualisations of HIV testing have shifted also; among men who have sex with men (MSM), testing can now be considered a normative behaviour, with strong pressure to test coming from other gay and bisexual men as well as from public health and voluntary sector organisations [8,9,10].

HIV self-testing (HIVST) is the latest in a long line of evolutions in HIV testing. Previously banned because of concerns surrounding potential harms, HIVST was legalised in the UK in April 2014, [11]. HIVST involves a person taking their own sample, processing their test, and interpreting their result. A positive result on an HIVST is the first step in a testing pathway; diagnosis requires undergoing further testing using a nationally recognised testing algorithm [4]. Free provision of HIVST has been patchy, with pilot and demonstration projects providing limited numbers of kits to specific risk groups in geographically defined areas [4,12].

### 1.2. SELPHI HIV Self-Testing RCT

SELPHI (An HIV Self-Testing Public Health Intervention) is the largest HIVST randomised controlled trial (RCT) in a high-income setting [13]. Between February 2017 and March 2018, SELPHI recruited 10,135 MSM (cisgender and transgender) and transgender women reporting anal intercourse with a man in their lifetime. SELPHI uses a one-off free HIVST intervention to assess whether an offer of HIVST can increase diagnosis rates of prevalent HIV infections, and an intervention comprised of a three-monthly offer of free HIVST to reduce the time between infection and diagnosis for incident infections [13]. Participants in SELPHI who receive HIVST kit(s) are provided with a BioSure^TM^ HIVST, which uses a whole blood sample. Intervention acceptability and test kit usability for SELPHI participants has been reported elsewhere [14,15].

SELPHI used the Behaviour Change Wheel (BCW), which includes the COM-B model of behaviour as an organising principle in conceptualising intervention components, their functions, and outcomes associated with each [16]. COM-B posits that alterations in capability, opportunity, and motivation are key to successful behaviour change interventions, and services can target alterations in these domains through careful intervention design [16,17].

Each domain of capability, opportunity, and motivation is divided into two sub-domains. Capability refers to physical and psychological abilities, while physical opportunity refers to environmental aspects shaping behaviour, and social opportunity refers to cultural factors constraining or facilitating engagement [16]. Motivation is divided into reflective and automatic processes (e.g. planning vs. emotions and impulses) [16].

The BCW comprises two levels of intervention: policy changes (e.g. legislation, regulation, communications, and marketing) and interventions directed at the individuals themselves (e.g. enablement, modelling, restriction). Table 1 presents an overview of intervention types with definitions.

The BCW provides recommendations for which intervention types are useful in targeting each behavioural domain (see Table 2) [16]. It provides a template from which to create theoretically informed behaviour change interventions which respond to specific needs.

COM-B and the BCW have been criticised for over-simplifying understandings of sources of behaviour and individual responses, and for their potential to ignore variation in need [18]. This could lead to the development of interventions which are insufficiently attentive to diversity amongst their intended users. However, COM-B and the BCW are used effectively across a wide variety of health conditions including HIV [17,19,20,21,22,23].

### 1.3. Understanding HIVST Interventions

Theorised to increase access for those with concerns related to privacy, convenience, and stigma, HIVST expands the locations in which an HIV test can be taken, and places increased emphasis on users’ responsibility to respond to the technology in specific ways [24]. Thought to be especially useful for those with less HIV testing experience because of the reduction in healthcare barriers [11,25,26,27], little is known about the ways in which individuals, or indeed groups, respond to the technology and incorporate it into health-seeking behaviours [4]. The majority of HIVST acceptability studies focus on the accounts of potential users before intervention exposure, and therefore do not reflect lived experience.

Interventions do not perform in uniform ways across individuals or groups [28]. Understanding how and why interventions function is key to optimising delivery processes, enhancing the potential to facilitate behaviour change by tailoring interventions for heterogeneous populations [7,28].

For HIV testing specifically, Flowers and colleagues urge increased consideration of the technological, psychosocial, and sociocultural context of HIV testing [7]. Changes related to sexual career and a person’s life-course are especially likely to affect how individuals seek and experience HIV testing interventions [7]. Those seeking HIV testing for the first time will likely have different needs than those who have more testing experience, factors to which intervention design should be attentive [7].

It is also important to better understand how interventions can be targeted based on group experiences to ensure optimal utility, especially from a life-course perspective. Self-testing provides the opportunity to design flexible interventions to meet a range of diverse needs, thus facilitating uptake in groups underserved by existing testing opportunities [7,11]. Further, it is important to understand how interventions impact on subsequent testing behaviour as this provides insights into the function they serve alongside a diverse set of other services.

### 1.4. SELPHI Logic Model

**Figure 1:**
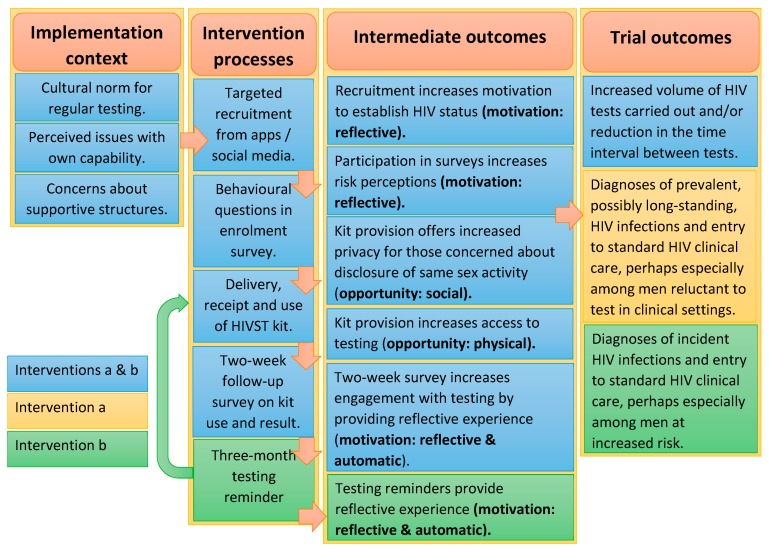
Logic model.

Likely pathways of impact from intervention delivery to the main trial outcomes are articulated through a COM-B informed logic model (Figure 1). An additional outcome of increasing the uptake of testing was also included, recognising the centrality of this aim to testing interventions. The logic model for SELPHI (Figure 1) draws from a systematic literature map, focus groups discussions (FGDs) with 47 MSM, and 17 key informant in-depth interviews (IDIs) [9,11,29,30]. As SELPHI is an RCT situated within a supportive policy environment, the framework focused on producing behavioural alterations through the intervention functions and their relationships with behaviour specified in COM-B (Table 1 and Table 2).

Contextual factors assumed to impact intervention delivery included a cultural norm for regular testing; perceived issues with capability and concerns about supportive structures.

The main testing barriers identified in formative work which HIVST could effectively respond to were motivation (reflective & automatic) and opportunity (physical & social). The intervention was designed to induce changes in these areas, while minimising concerns around capability (psychological & physical), which presented the primary barrier to intervention implementation. We sought to ameliorate capability issues through intervention design.

**Figure 2:**
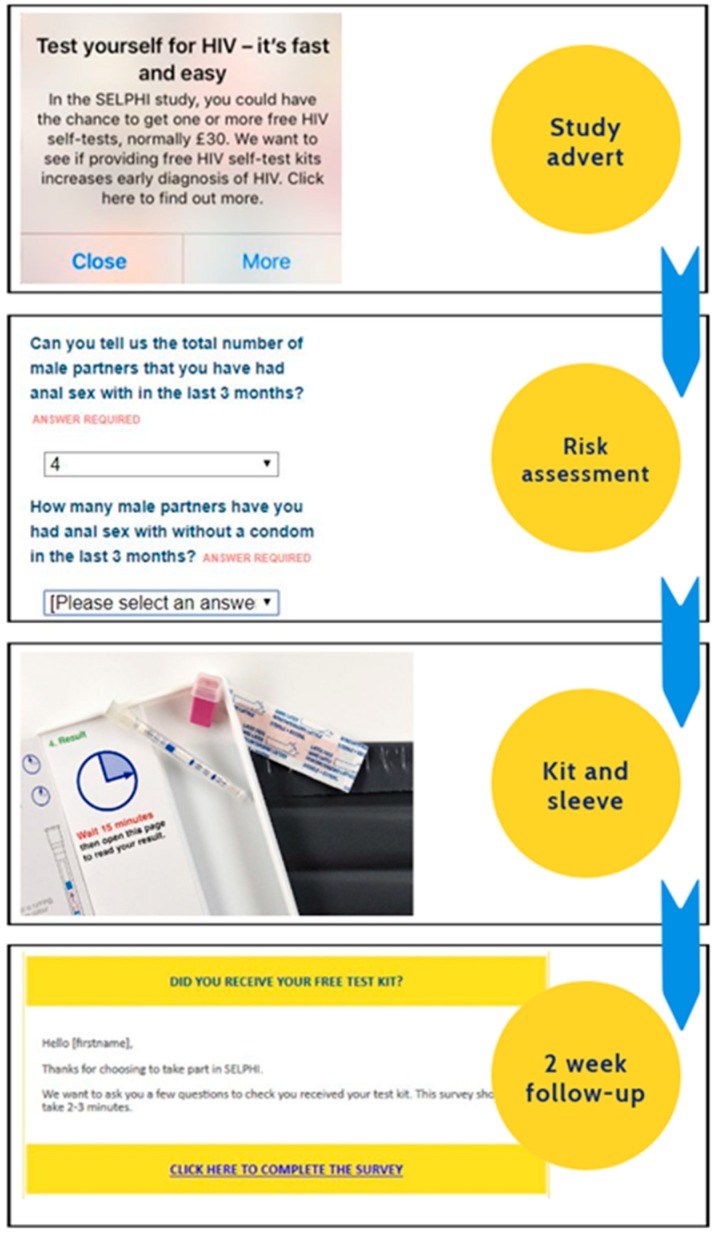
Intervention A.

Intervention A (Figure 2) is linear, moving from targeted recruitment to a risk assessment represented by behavioural questions in a survey, then to the receipt and use of the HIVST, and lastly a two-week follow-up survey. The advertisement was classified as a form of education and persuasion, seeking to enhance motivation (reflective). The risk assessment was a form of persuasion, enhancing motivation (reflective) by increasing feelings of vulnerability to HIV. Free kit provision was a form of enablement increasing opportunity (physical). The two-week follow-up was a form of enablement, increasing motivation (reflective & automatic). In the SELPHI pilot, participants reported the test kit was easy to use, that the instructions were simple and the support structures adequate for their needs [14,15], demonstrating that our efforts to ameliorate capability issues through intervention design were successful.

**Figure 3:**
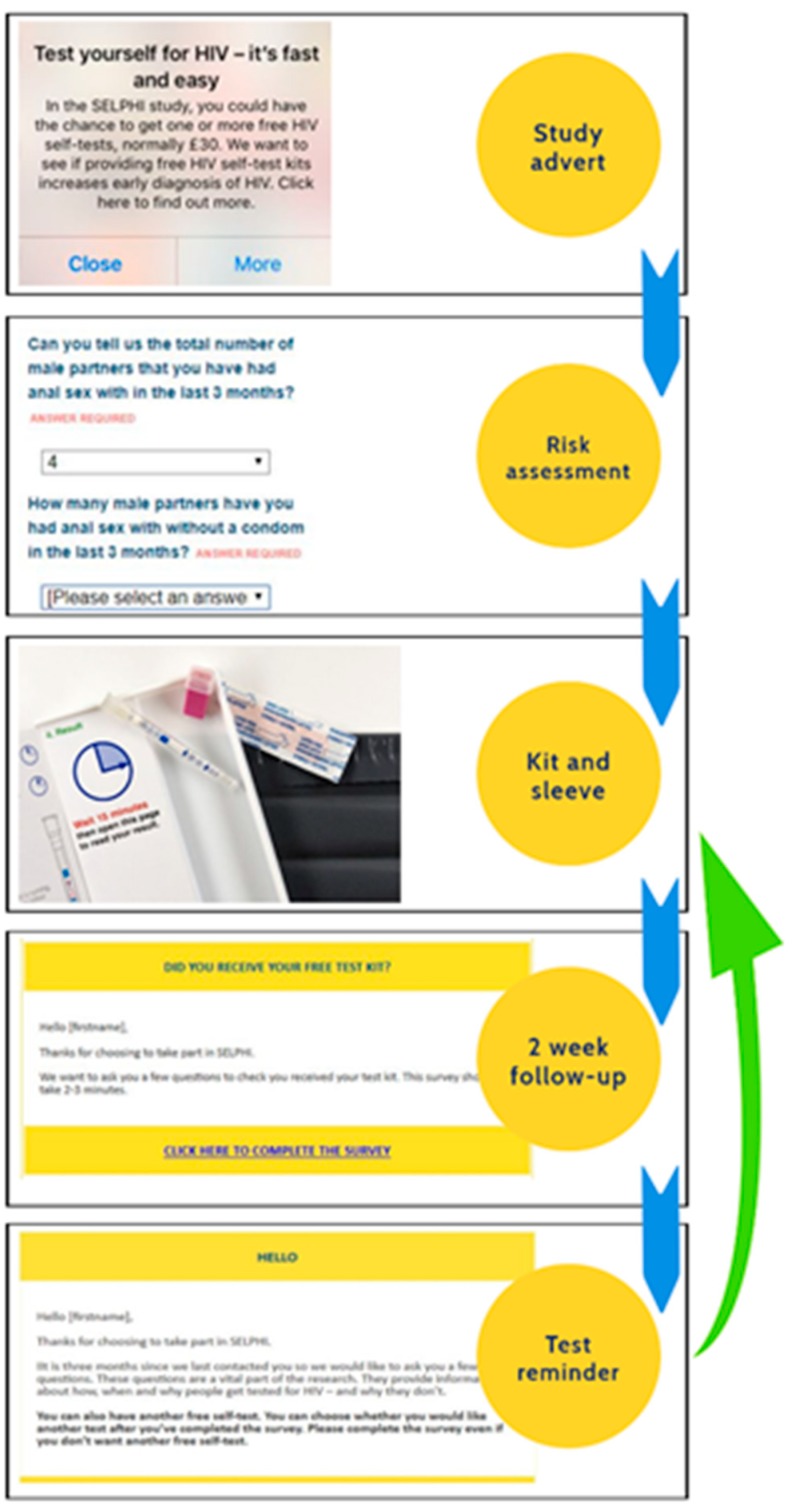
Intervention B.

Intervention B (Figure 3) includes all elements of A, with additional cyclical components consisting of a testing reminder and a linked risk assessment delivered every three months which, should a kit be requested, triggers delivery of a new HIVST kit and another two-week follow-up survey. The testing reminder and linked risk assessment were forms of persuasion, targeting motivation.

The logic model articulates intermediate outcomes assumed to be precursors to achieving the trial outcomes. Each intermediate outcome relates to a documented deficit or need constraining testing within the target population and which intervention design has identified as a key area which requires change. They are described as an outcome (e.g. increased risk perception) and a COM-B-linked domain which required alteration to achieve this. None of the intermediate outcomes were related to capability concerns as a barrier to uptake as these had already been mitigated via the provision of enhanced support and through intervention messaging [14].

Understanding differences in experience in groups of individuals has long been a public health aspiration. Latent class analysis is a method often used in quantitative studies which seeks to create groups based on shared demographic and behavioural traits and uses these to investigate differences in outcomes [31,32]. We draw on the foundational principles of this approach and apply them to a qualitative analysis to examine how different groups of participants understand the utility of HIVST in the context of their testing behaviour. These findings can be used to guide commissioners and health promoters in how HIVST interventions might be adapted to better meet a range of needs and improve outcomes.

This paper aims to explore how the SELPHI interventions might be experienced by, and the pathways to impact on behaviour for, different groups of RCT participants. The specific objectives are: to develop a participant typology to identify commonalities in the intervention experience and how these vary according to sexual and testing behaviours; to describe how interventions can be designed to respond to these commonalities; and to examine the utility of COM-B as a model for understanding behaviour change in relation to HIVST.

## 2. Methods

### 2.1. Participants and Procedures

This study involved 37 in-depth semi-structured interviews with purposively sampled cis-gender MSM. The initial 10 interviews were conducted in May 2017, with a further 27 taking place between January and October 2018. Ethical approval was sought and granted from University College London [ref: 11945] and London School of Hygiene & Tropical Medicine [ref: 9233/001].

Interviews were conducted face-to-face (n = 20) and remotely (n = 17). Purposive sampling was used to ensure a diversity of participants, based primarily on testing history and then on geographical location, highest educational qualifications, age, and ethnicity. Interviews were conducted in Cardiff, Newcastle, and London, ensuring participation from men in areas with variable health service development and differences in the prominence of gay scenes. Two interviews were conducted with participants who had a positive result from HIVST.

A topic guide for interviews was developed drawing from earlier formative work, the logic model, and the broader HIVST literature [9,11,26,33]. The first iteration covered testing history, engagement with SELPHI, intervention experiences, and preferences for future HIVST development. After piloting, this was refined. Following the first ten interviews the topic guide was expanded to provide additional focus on the HIVST intervention being used in the RCT. Questions about trial infrastructure and intervention processes were added; participants were asked to revisit the adverts they were recruited from and the surveys they filled out and take time reviewing components of the kit. Interviews were audio recorded and transcribed verbatim.

### 2.2. Analytic Procedures

A deductive framework approach to data analysis was chosen to focus on specific elements of the intervention and because of the need to compare these across participants [34]. Key domains of enquiry were extracted from the topic guide, the interviews, and the broader HIVST literature and organised around broad themes. This analysis draws from a section of this framework which focuses on intervention function. Each intervention component and the intermediate outcomes, assumed to be a result of these, had their own code to which data were assigned by the first author. COM-B domains were mapped onto the framework to ensure it captured relevant emerging data. The analysis remained attentive to the emergence of new issues and lines of enquiry, especially around the groupings of participants. The framework itself was refined twice: once after two interviews in the pilot phase, and again following the addition of new questions in the second phase of data collection.

During analysis it emerged that participants were clustered qualitatively into three main groups based on their life-course, HIV testing history, and engagement with HIVST. We solidified this into a typology of participants based on prior testing and orientation to SELPHI, and then looked to see whether accounts of intervention experiences and perceived intermediate impacts differed among the groups identified in the typology, and how these aligned with constructs from the logic model. This was then qualitatively assessed for strength and clarity of feeling expressed by intermediate outcome for each of the groups. These themes relating to intervention functions by participant groups were reviewed by TCW, PW, AJR, and FMB to ensure coherence of interpretation and agreement with assessments related to strength of evidence.

## 3. Results

The SELPHI interventions were experienced variably by the groups identified in our analysis. In this section, we first outline the groups and their defining characteristics, before describing each intervention component and associated intermediate outcomes. The differences in intermediate outcomes across groups are then presented.

### 3.1. A Testing Typology 

In our analysis, we found three main groups who had distinct experiences of intervention performance: inexperienced testers (n = 14), pro self-testers (n = 11), and opportunistic adopters (n = 12). These groups were classified primarily based on their testing history and motivations for engaging with HIVST. These groups represent individuals at different stages of their life-course and sexual careers; they are therefore fluid and exist on a spectrum. Although diversity of experience existed within these groups, their shared characteristics meant that the interventions functioned in differing ways for these three groups. These distinct experiences are useful for understanding how intervention components varied in their utility and the potential for future intervention optimisation. Table 3 presents our sample demographics.

#### 3.1.1. Inexperienced Testers

This group’s defining characteristic is their estrangement from testing services. Inexperienced testers had either never previously tested or had not tested in quite some time. These men were usually at the beginning of their sexual careers, but also included those who had been sexually active for many years but had never routinely tested for HIV. All were below the age of 25 or over the age of 40. This group often had profound issues relating to stigma, shame, internalised homophobia, and traditional conceptualisations of masculinity. HIVST facilitated engagement with testing due to the reduction in barriers which had previously prevented them from testing. Barriers for this group clustered around the COM-B domains motivation (reflective & automatic); opportunity (social & physical) and capability (psychological). These barriers included psychological and social barriers such as stigma, perceived lack of privacy, and low self-efficacy regarding clinic access, as well as structural barriers such as geographic isolation from existing testing services. Some inexperienced testers viewed testing as a normative behaviour, but these views were much less strongly held than in the other groups. If they tested at all, individuals in this group usually tested in response to risk, with a small minority testing for reassurance. None tested out of routine.

#### 3.1.2. Pro Self-Testers

Men in this group had an established testing routine but tested sub-optimally based on guidelines or less than their own preferences because of barriers to accessing services. Generally speaking, these men could be understood to be at an intermediate point of their sexual careers. They may have been sexually active for some time but often retained barriers to living very open lives related to stigma, shame, conceptualisations of masculinity, and homophobia. They were spread across all age groups. These barriers related to the COM-B domains of opportunity (social & physical) and capability (psychological). Barriers to testing services faced by this group included stigma, privacy concerns, and perceptions that services were inaccessible or inappropriate. This group experienced a tension between norms to test balanced against their personal barriers to access which constrained their testing behaviour, sometimes leading to feelings of guilt, shame or of being stigmatised. HIVST helped to resolve these barriers and facilitate testing uptake thereby alleviating negative feelings about not testing. HIVST was the preferred testing option and they used HIVST when testing in response to risk, for reassurance and out of routine.

#### 3.1.3. Opportunistic Adopters

The final group in our analysis were already very engaged with testing services where their needs were generally well met. This group had no discernible COM-B barriers, except for some minor issues with physical opportunity related directly to accessibility of clinics based on opening or waiting times. Opportunistic adopters tended to test at least annually, but often much more frequently. Men in this group were, on the whole, comfortable with their sexual orientation, led quite open lives, and had access to services which met their needs in an appropriate way. These men were all over 25 years of age. Although HIVST uptake was facilitated by increased convenience; they had no obvious deficits to which HIVST responds. They used self-testing largely out of novelty, as a replacement for a routine test or to gain reassurance.

### 3.2. Intervention Components and Behavioural Influences

**Figure 4:**
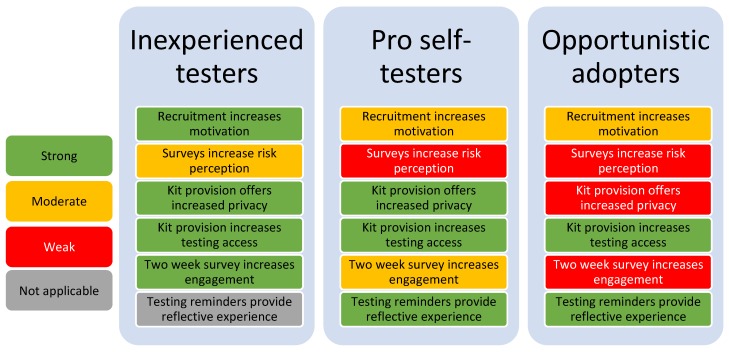
Outcomes and evidence strength by group.

We found key differences in the way the intervention functioned for each group. Inexperienced testers’ experiences matched most closely with the hypotheses in our logic model. Pro self-testers experiences diverged, especially around elements designed to enhance motivation (automatic & reflective). Opportunistic adopters diverged further, with significantly different experiences when compared to inexperienced testers. Figure 4 presents differences across groups of SELPHI participants by assumed intermediate outcome.

#### 3.2.1. Recruitment and Testing Motivation

The SELPHI logic model assumed that the adverts used to recruit participants would increase motivation to test. These adverts were a form of education and persuasion targeting reflective motivation. Evidence relating to this intermediate outcome was mixed; while strong evidence supporting this hypothesis was found for inexperienced testers, evidence was moderate for the other two groups.

For inexperienced testers, the messages used in advertising functioned largely as expected, producing the assumed outcome. They increased motivation by highlighting a convenient, free testing opportunity, and provided a reflective experience where individuals could consider the process of testing and the benefits it could bring.

Deep down, I knew that it was something that I should do anyway. And it was free. And I knew that not all people would get it. But it was free if I was selected. And it just seemed like I’d no excuse, really, to turn it down. It’s free. It would be in private. I wouldn’t have to go to the clinic. And I’d get a little bit of peace of mind if it came back negative. (23-year-old man, undisclosed sexual orientation, not previously tested, inexperienced tester).

Pro self-testers had more mixed response to the recruitment element. For some the SELPHI adverts were profoundly motivational and functioned as expected, while for others these simply came at an appropriate time.

For me, it was timing. It was just a case of like, ‘okay, it’s been a year’; I knew I had had some…maybe not the cleverest of sexual encounters a couple of months beforehand, and it literally landed at the right time in my thought processes so I was like ‘yep, we’ve got that whole World AIDS Day thing coming up again, and this is probably about the right time to get tested again’. (29-year-old gay man, tested in last 12 months, pro self-tester)

Opportunistic adopters rarely identified the adverts as increasing motivation to establish their HIV status, largely because their motivation was already high, and they did not have a COM-B barrier to address in this regard. The majority stated that the adverts came while considering or planning their next test. For the minority who found this element motivational, the source was the novelty of HIVST.

#### 3.2.2. Surveys and Risk Perception

The SELPHI logic model assumed that participating in surveys pertaining to risk behaviour would increase risk perception, and these were considered part of the intervention itself. These surveys were considered a form of persuasion, working through reflective motivation pathways in the COM-B model. Overall, this intervention component had the weakest evidence across all MSM. Some inexperienced testers and a minority of pro self-testers felt the surveys functioned as assumed, while opportunistic adopters felt they did not.

Most inexperienced testers anticipated that the types of questions asked in the risk assessment would form part of the intervention. Although some found answering these personally uncomfortable because they highlighted sexual risk, others externalised this feeling to ‘others’ who might find this element of the intervention challenging. This externalisation was also present for a small number of pro self-testers, although this group on the whole did not report this intervention component increased motivation.

Some of the questions […] I felt might make some people worry, maybe […] The ones relating to having sex without protection. Also, having sex whilst taking drugs as well. That was another one. And I thought, ‘oh, okay, well, that’s half of gaydom’. (49-year-old man, undisclosed sexual orientation, tested in last 12 months, pro self-tester).

Opportunistic adopters universally felt the questions were unproblematic and expected. Experience was a key component of this lack of intervention impact: this group identified being asked similar things routinely while accessing other testing services. This was also related to length of time openly identifying as gay or bisexual and a feeling of self-acceptance and acceptance of ones’ own risk behaviour.

It doesn’t make me feel any different about myself. Because I have been actively gay for 20 years now. Maybe or definitely, in my early years, that would make me feel uncomfortable that I was promiscuous, so to speak. But having been active for 20 years now, it’s not something that bothers me anymore. (40-year-old gay man, tested in last 12 months, opportunistic adopter)

#### 3.2.3. Kit Provision and Privacy to Test

The provision of the HIVST was assumed to increase social opportunity by providing additional privacy for testing. This intervention approach is termed enablement in the BCW. Evidence for this intermediate outcome was generally strong overall, with inexperienced testers and pro self-testers identifying the intervention as performing in this way. For opportunistic adopters, however, there was weak evidence for this outcome.

Inexperienced testers and pro self-testers often primarily engaged with SELPHI because of the reduced psychosocial barriers to clinic access including stigma, shame, and a lack of privacy. Unsurprisingly, for these two groups the increased privacy offered by HIVST was incredibly valuable, dramatically increasing social opportunity. For some this was related strongly to keeping their sexual identity private or hidden while also accessing services, while for others this was out of a preference for privacy and control while also meeting norms around HIV testing.

The SELPHI thing actually appealed to me that I could be totally anonymous. […] I’ll say the shame and embarrassment. And I’ll put it that way because I’m in the closet and probably prefer to remain that way. So, it is the difficulty of taking that step [accessing a clinic]. […] (55-year-old bisexual man, not previously tested, inexperienced tester)

Opportunistic adopters did not have privacy concerns when accessing services, partly because strong social norms supporting HIV testing did not conflict with desires for privacy or anonymity.

#### 3.2.4. Kit Provision Increases HIV Testing Access

Providing HIVSTs at no cost was assumed to increase physical opportunity by reducing testing barriers related to inconvenient or geographically removed HIV testing services. Again, this is classified as enablement in the BCW system. This was the only assumed intermediate outcome on which there was positive evidence across the three groups. How important this was to each of the groups differed, however.

Inexperienced testers identified the increases in physical opportunity as being helpful in facilitating testing. This was not necessarily the area in which they had the most pronounced need as the privacy offered by HIVST was more important. Rather, this was secondary or incidental.

I started having sex with other men more frequently. And, at that point, I started to consider [testing for HIV]. And that was around the time when... about a month or so after that when, I think, the SELPHI thing came up on Grindr. And that’s when I thought, mmm, self-testing seemed like a better idea, I thought. (23-year-old man, undisclosed sexual orientation, not previously tested, inexperienced tester)

For the majority of pro self-testers, although this was important, it was also secondary to the increased privacy provided by the intervention which was the main benefit. For some however, their predominant testing barriers were related to inconvenient or geographically removed HIV testing services. For these men, HIVST met their needs in terms of providing a physical opportunity.

But when I had seen it, and doing it at home I thought it was a great idea, because you’re at home, you can sit and get your result there and then, you can take it from there, and go and get support if you needed to. (30-year-old gay man, tested in last three months, pro self-tester)

For opportunistic adopters, the increased convenience was the main element of the intervention that functioned as assumed for this group. Increased convenience, and therefore physical opportunity, was the primary intermediate outcome experienced by these men.

So, I think for me, whenever I found out about this I was like, ‘Okay, well that makes my life a lot easier,’ because I work long hours and I have quite an unpredictable job. So, trying to maybe get time off and having that time off assured so that I can go to an appointment is quite difficult, so this study was very helpful. (27-year old gay man, tested in last three months, opportunistic adopter)

#### 3.2.5. Two-Week Survey and Testing Engagement

Our logic model assumed that the two-week follow-up survey (which we envisage would remain an integral part of the intervention if rolled-out) increased engagement with HIV testing generally through reflective and automatic motivation channels while also providing support. Overall, this intermediate outcome had the most inconsistent evidence across all groups and accounts diverged dramatically. Although more inexperienced testers felt that this element was supportive and provided a reflective experience, for pro self-testers this was inconsistent. For opportunistic adopters this was rarely the case.

Inexperienced testers were more likely than the two other groups to identify the two-week follow-up as providing support and being a reflective experience. For these participants, it was an important part of the intervention which provided a degree of connection to the study team which was valued. This feeling of connection itself provided a prompt to reflect on trial involvement and intervention experiences.

I’ve done the test and then there was nothing for a while, and then you get the survey back and you feel, ‘Oh yes, they are still thinking about me. I am still part of this study, I still feel included.’ Whereas I think if it’d just been left and there was nothing you have thought, ‘Well, okay, I’ve got a free test out of it but what’s the point?’ (35-year-old gay man, tested in last 12 months, inexperienced tester)

Neither of the inexperienced testers who received a positive result noticed the delivery of, or filled in, the survey, so could not comment on its value.

Pro self-testers on the whole thought the provision of the two-week survey was a positive element, and some valued the support, anticipating that some people would receive a positive result. For the majority however, this element did not provide a reflective experience and did not increase personal engagement with testing. This is perhaps a function of strong testing norms, but also high psychological and social barriers to testing in this group which create ambivalence around testing. One participant who expressed significant ambivalence about the survey said:

Interviewer: So, if you were getting the test from the NHS, you wouldn’t necessarily want to fill out one of those surveys?

Participant: I wouldn’t want to, but I think it would be good practice to. If you were going to distribute self-tests, it would be good to use a follow-up. If in my situation now, if I wasn’t studying it, if it was just a routine thing and it was negative, I probably wouldn’t fill it out, it’d go into the junk box. But I still think it’s probably a good thing to do. (32-year-old gay man, tested in last 12 months, pro self-tester).

Opportunistic adopters nearly universally felt that the two-week survey was part of a trial process rather than a supportive structure. It was not a helpful component of the intervention for them, although there was some acknowledgment that as a reflective experience it could be helpful for others.

I took it as a study. I felt like I was trialling something, I didn’t have any expectations of what it would be. Nothing stood out to me, but again, with my sexual health and dealing with sexual health services, there is a resilience or comfortability of having to share information, if that makes sense? (25-year-old gay man, tested in last 12 months, opportunistic adopter)

#### 3.2.6. Testing Reminders and Reflection 

Testing reminders with a linked risk assessment were assumed to provide a reflective experience for those receiving repeat offers of HIVST kits in intervention B, thus enhancing motivation to test. Because of the cyclical, repeated nature of the intervention, inexperienced testers either transitioned to being pro self-testers or opportunistic adopters.

In intervention B, testing reminders were acknowledged by all to function as assumed by providing a prompt to consider sexual behaviour and HIV risk in the intervening three months. This had multiple effects: it normalised HIV testing, increased risk perception, provided a prompt to reflect on past testing, and increased motivation to test again.

Just made me think a bit harder of the past three months, what I’d been doing, yeah. It didn’t make me feel anything like I shouldn’t be asked this or this and that, yeah, it was just a questionnaire and it just made me think about all the movement I had in the last three months [...] (27-year-old bisexual man, tested in last six months, pro self-tester)

## 4. Discussion

This qualitative study investigating intervention performance with 37 cis-gender MSM participants in the SELPHI RCT identified three main participant groups, defined by their testing history and engagement with HIVST, who had distinct intervention experiences and intermediate outcomes.

Inexperienced testers largely had not been previously tested, or were disengaged from testing because of profound barriers to service access. Pro self-testers had a testing history, and sometimes had a routine, but their frequency of testing was constrained by many of the same barriers as inexperienced testers. HIVST resolved many of these barriers for both groups, leading to expectations of increased testing frequency. Opportunistic adopters were very well served by existing services and had a routine involving frequent testing. For this group, HIVST uptake was facilitated by the novelty of the technology, or because of increased convenience.

Intervention performance across these groups was highly variable. For inexperienced testers, intermediate outcomes were closest to the six assumptions in the logic model. Divergence began for pro self-testers and was most pronounced for opportunistic adopters. Divergence was found to be related to the pre-existing needs of the groups and their corresponding testing barriers. Inexperienced testers had the most pronounced needs and therefore the most favourable outcomes, whereas for opportunistic adopters who had less needs (mainly related to physical opportunity), the intervention components assumed to impact on the other domains had little relevance. The exception to this was testing reminders, which impacted all groups in a similar way increasing reflective motivation.

### 4.1. Strength and Limitations

This is the first systematic study of the relationships between intervention components and their influences on uptake of HIVST. Some limitations are noted. Our sample was derived entirely from an RCT which had substantial informed consent procedures, providing a level of detail about the kit and trial processes beyond what would be provided in standard free HIVST provision. Those who engaged may be more likely to have altruism as a primary motivator indicating that this group may not be reflective of those who have need for HIVST [14]. In addition, a requirement of participation was to provide a mailing address and consent for the study team to link to clinic records and monitoring data held by the government. This may have dissuaded those with the highest privacy barriers from participating, a group with pronounced utility for HIVST [11,26,33].

The sample was relatively small (n = 37). Conducting additional interviews with larger numbers of participants may have led to a refinement of groups with larger amounts of data. In addition, as HIVST becomes more mainstream over time and more gain self-testing experience, these groups may also shift. This should therefore be viewed as indicative of a broad typology of MSM that are potential beneficiaries of HIVST prior to widespread free availability in the UK.

Our results will be applicable to other culturally similar settings with comparable health services. They may not effectively translate to very different contexts such as the USA and Southern Africa where motivations to use HIVST will vary along with intervention approaches.

Finally, our analysis relies on participants accurately reflecting on their thoughts and feelings during intervention engagement some time afterwards, potentially not fully reflecting their experiences.

### 4.2. COM-B, HIVST, and Challenges for the Model

Our analysis poses some challenges for the application of COM-B as an overarching framework. We required a more nuanced framework of motivation to analyse the influence of social norms drawn from peers, community, and society and the way that they might shape engagement with interventions. Indeed, although aspects of social norms are captured in motivation, the centrality of these to behaviour is perhaps not fully accounted for in the existing model. This is a challenge for understanding the experiences of opportunistic adopters especially; many had engaged because of strong norms to test frequently and because they had a routine to do so—a well-documented phenomena and one which HIVST has been hypothesised to respond to among MSM in high income settings [8,9].

This is a critical issue for HIV prevention research where norms are central to uptake of precautionary behaviours and shape use of condoms, testing and biomedical prevention technologies [8,9,10,35,36,37]. The model should pay additional attention to the impact on motivations of strong social norms arising from peers, service providers, and the wider community [18,38,39].

### 4.3. Considerations for Future HIVST Intervention Development

Life course relating to sexual experience and acceptance of sexual identity clearly have a significant impact on how the SELPHI intervention was experienced. This demonstrates the potential for intervention optimisation available for those seeking to implement HIVST. Advertising can be designed for those with most utility for HIVST: inexperienced testers and pro self-testers, targeting key influences revealed by our COM-B analysis. A brief risk assessment, sexual history, and testing history could then be taken, with an automated process streaming service users into specific intervention types based on their characteristics, preferences and histories. The two-week results survey can be conceptualised as playing a dual role of maintaining engagement in testing for those with negative results, while also offering support for those with positive self-tests. Enhanced interventions providing additional support could then be provided for inexperienced testers and pro self-testers with lower level support provided for those with more testing experience. This could make interventions more acceptable and more efficient by providing a more personalised HIVST service.

It is important to note that men did not have static positions within these categorisations. For some, engagement with the intervention facilitated a movement from one group to another. This was especially true for inexperienced testers who tended to move to being pro self-testers or opportunistic adopters following intervention exposure. Intervention design should therefore be attentive to shifts within individuals who use HIVST, especially if longer-term provision will allow repeat testing. HIVST service provision should also be attentive to the potentially transformative properties of self-testing, especially for those who face barriers to accessing other testing services.

The SELPHI logic model can be refined as a result of this analysis, clarifying behavioural influences and their implications for the interventions. Doing so will also enable the inclusion of an additional level of assumed outcomes between intermediate and ultimate (trial) outcomes clarifying the likely pathways to these.

## 5. Conclusions

This study demonstrates the profoundly different experiences MSM can have when engaging with HIV testing interventions. Clearly, adaptations can be made to better meet the needs of individuals based on needs related to their testing history, life course, and sexual careers. Further, we provide a critique of COM-B, demonstrating limitations in its application when considering engagement in some groups for which a more elaborated theory or model of motivation is required.

## Figures and Tables

**Table 1:** COM-B intervention types and definitions (reproduced from Michie et al., 2011, with permission).

Interventions	Definition
Education	Increasing knowledge or understanding
Persuasion	Using communication to induce positive or negative feelings or stimulate action
Incentivisation	Creating expectation of reward
Coercion	Creating expectation of punishment or cost
Training	Imparting skills
Restriction	Using rules to reduce the opportunity to engage in the target behaviour (or to increase the target behaviour by reducing the opportunity to engage in competing behaviours)
Environmental restructuring	Changing the physical or social context
Modelling	Providing an example for people to aspire to or imitate
Enablement	Increasing means/reducing barriers to increase capability or opportunity ^1^

^1^ Capability beyond education and training; opportunity beyond environmental restructuring.

**Table 2:** Relationship between COM-B domains and interventions (reproduced from Michie et al., 2011, with permission).

Model of behaviour: sources	Education	Persuasion	Incentivisation	Coercion	Training	Restriction	Environmental restructuring	Modelling	Enablement
**C-Ph**					√				√
**C-Ps**	√				√				√
**M-Re**	√	√	√	√					
**M-Au**		√	√	√			√	√	√
**O-Ph**						√	√		√
**O-So**						√	√		√

**Table 3:** Sample demographics.

**Ethnicity**	White	29
	Black	3
	Asian	2
	Other/mixed	3
**Sexual orientation**	Gay	24
	Bisexual	5
	Don’t use a term	1
	Undisclosed	7
**Recency of HIV testing**	Never tested	7
	12 months +	17
	<12 months	13
**Highest education qualification**	Low ^1^	7
	Medium ^2^	11
	High ^3^	19
**Condomless anal intercourse partners in preceding three months**	0	12
	1	14
	2–3	7
	4–10	4
	10+	0
**Test outcome**	Positive	2
	Negative	35
**Intervention**	Intervention A	27
	Intervention B	10

^1^ GCSEs and below. ^2^ A-levels or equivalent, higher education below degree level. ^3^ Degree or higher.
